# Use of Bevacizumab in recurrent glioblastoma: a scoping review and evidence map

**DOI:** 10.1186/s12885-023-11043-6

**Published:** 2023-06-14

**Authors:** Minjie Fu, Zhirui Zhou, Xiao Huang, Zhenchao Chen, Licheng Zhang, Jinsen Zhang, Wei Hua, Ying Mao

**Affiliations:** 1 Department of Neurosurgery, Huashan Hospital, Shanghai Medical College, Fudan University, #12 Middle Urumqi Road, Shanghai, China; 2 National Center for Neurological Disorders, Shanghai, China; 3 Shanghai Key Laboratory of Brain Function and Restoration and Neural Regeneration, Shanghai, China; 4 Neurosurgical Institute of Fudan University, Shanghai, China; 5 Shanghai Clinical Medical Center of Neurosurgery, Shanghai, China; 6 Radiation Oncology Center, Huashan Hospital, Shanghai Medical College, Fudan University, Shanghai, China; 7 Department of General Surgery, Huashan Hospital, Shanghai Medical College, Fudan University, Shanghai, China

**Keywords:** Bevacizumab, Recurrent glioblastoma, Combined therapy, Quality of life

## Abstract

**Background:**

Glioblastoma (GBM) is the most malignant primary tumor in the brain, with poor prognosis and limited effective therapies. Although Bevacizumab (BEV) has shown promise in extending progression-free survival (PFS) treating GBM, there is no evidence for its ability to prolong overall survival (OS). Given the uncertainty surrounding BEV treatment strategies, we aimed to provide an evidence map associated with BEV therapy for recurrent GBM (rGBM).

**Methods:**

PubMed, Embase, and the Cochrane Library were searched for the period from January 1, 1970, to March 1, 2022, for studies reporting the prognoses of patients with rGBM receiving BEV. The primary endpoints were overall survival (OS) and quality of life (QoL). The secondary endpoints were PFS, steroid use reduction, and risk of adverse effects. A scoping review and an evidence map were conducted to explore the optimal BEV treatment (including combination regimen, dosage, and window of opportunity).

**Results:**

Patients with rGBM could gain benefits in PFS, palliative, and cognitive advantages from BEV treatment, although the OS benefits could not be verified with high-quality evidence. Furthermore, BEV combined therapy (especially with lomustine and radiotherapy) showed higher efficacy than BEV monotherapy in the survival of patients with rGBM. Specific molecular alterations (IDH mutation status) and clinical features (large tumor burden and double-positive sign) could predict better responses to BEV administration. A low dosage of BEV showed equal efficacy to the recommended dose, but the optimal opportunity window for BEV administration remains unclear.

**Conclusions:**

Although OS benefits from BEV-containing regimens could not be verified in this scoping review, the PFS benefits and side effects control supported BEV application in rGBM. Combining BEV with novel treatments like tumor-treating field (TTF) and administration at first recurrence may optimize the therapeutic efficacy. rGBM with a low apparent diffusion coefficient (ADCL), large tumor burden, or IDH mutation is more likely to benefit from BEV treatment. High-quality studies are warranted to explore the combination modality and identify BEV-response subpopulations to maximize benefits.

## Introduction

Glioblastoma (GBM) is the most aggressive type of primary malignant tumor of the brain in adults [1]. Despite the new combination of Stupp protocol, including radiation and chemotherapy with maximal surgical resection and tumor-treating field (TTF), the prognosis remains unsatisfactory as most tumors recur in situ [1, 2]. Several interventions, including targeted therapies, have been attempted to improve the prognosis of GBM. As GBM is a hyperemic tumor involving the upregulation and activation of VEGFA and HIF [3], VEGFA is a reasonable target molecule in the treatment of GBM. Bevacizumab (BEV), a humanized monoclonal antibody inhibiting VEGFA, was considered a promising candidate for treating GBM, given its clinical benefits in other cancers such as colorectal cancer [4], renal cell carcinoma [5], non-squamous non-small cell lung cancer [6], and cervical cancer [7]. Success in the treatment of other tumors persuaded researchers to conduct phase III AVAglio and RTOG 0825 clinical trials in patients with newly diagnosed GBM. However, both clinical trials didn’t improve the overall survival (OS) in the BEV treatment arm. Further, a randomized phase II TAVAREC clinical study demonstrated that BEV treatment had no significant improvement on progression-free survival (PFS) and OS in Grade 2 and Grade 3 gliomas [8]. A phase III trial by Wick et al. did not find any OS benefits with combined therapy of BEV plus lomustine, compared with lomustine alone [9]. Based on these several clinical trials, BEV is considered ineffective in prolonging OS for recurrent GBM (rGBM) by the European Association Neuro-Oncology (EANO) [10, 11]. Nevertheless, clinical benefits other than the prolongation of survival were possibly observed. The EORTC protocol demonstrated that BEV decreased steroid dependence and relieved para-tumor edema in patients with GBM [8]. Despite a lack of evidence supporting its ability to prolong OS, BEV was approved by the FDA (U.S. Food and Drug Administration) in 2009 as a treatment for rGBM and was included in the 2021 EANO guidelines due to its demonstrated improvement in quality of life and safety [11].

BEV might not be suitable for the treatment of all rGBM patients in general based on the outcome of these randomized trials. In the AVAglio trial, subgroup analysis revealed that the TCGA-proneural GBM subtype had an OS benefit from the administration of BEV. Further, epigenetic mechanisms could also influence the sensitivity of BEV, as demonstrated by Cloughesy et al.‘s finding that methylguanine-DNA methyltransferase (MGMT) methylation may be predictive for onartuzumab (ONA) + BEV outcomes in GBM. It is necessary to perform subgroup analyses to specifically identify the survival benefits of the treatment of BEV. However, no consensus has been reached regarding the subset of rGBM patients who are sensitive to BEV. Furthermore, the optimal combination therapy, dosage efficacy, and correct indication for BEV therapy are still controversial.

Given the considerable uncertainty surrounding BEV treatment strategies, we aimed to systematically review the current evidence associated with BEV therapy by mapping evidence. We aimed to answer the following five questions: (1) Could BEV-containing regimens bring survival benefits to patients with rGBM, compared with non-BEV treatment regimens? (2) Could BEV combined with other therapies prolong the OS of patients with rGBM, compared with BEV monotherapy? (3) Could BEV treatment improve quality of life (QoL) and reduce the adverse events (AEs) in rGBM? (4) Could some subgroups harboring specific clinical or molecular characteristics gain survival benefits from BEV treatment? (5) What are the optimal dosages and indications for the BEV treatment in rGBM?

## Methods

### Search strategy and study selection

The scoping review and mapping evidence were conducted following the PRISMA extension for scoping reviews [12]. A comprehensive literature search was performed in electronic databases including PubMed, Embase, and the Cochrane Library, on March 27th, 2022.

#### Inclusion criteria

(1) Patients with recurrent high-grade glioma (WHO grades 3–4) or GBM (WHO grade 4), regardless of age, gender, or pathological type; (2) Patients who were treated with BEV alone or in combination. Treatment types were focused on but were not limited to BEV alone, or BEV plus radiotherapy, chemotherapy (including carmustine implants), chemoradiotherapy, surgery, immunotherapy, and TTFs; (3) The outcomes of interest included OS, PFS, QoL, and AEs (cerebral edema and cognitive deficits) incidence; (4) Study types included randomized controlled trials (RCT), case-control trials (CCT), observational trials, pre- and post-control studies, and systematic reviews.

#### Exclusion criteria

(1) Case reports and conference abstracts; (2) Protocols but not reports of the study result; (3) Studies that were not reported in English.

Two reviewers independently screened the titles and abstracts of the retrieved records. Following the initial screening, full texts of the trials that passed title/abstract screening were scrutinized to confirm eligibility for the analyses. Disagreements were resolved by discussion with a third person if necessary. A PRISMA flow diagram was constructed to show the full article-selection process.

### Data extraction

Two authors (Minjie Fu and Xiao Huang) independently examined the studies and extracted data using a standardized spreadsheet with the following characteristics: trial type, number of participants, type and administration of interventions, the definition of outcomes, measurement variables, and key findings. In situations of discrepancies, the third author (Zhirui Zhou) was consulted for final decision-making.

### Data coding and definition

Selected studies were coded according to the type and administration of interventions (BEV monotherapy or BEV combined therapy). Classification criteria were discussed by the professional group. The term “Beneficial” was defined as a finding that had one or more of the following results: prolonged OS or improved QoL or PFS. The term “Harm” was defined as a finding that had one or more of the following results: decreased OS or PFS or worse QoL. The term “No difference” was defined as no significant difference or no difference reported between the groups for OS, PFS, or QoL. “Inconclusive” was defined as a finding that demonstrated both beneficial and harmful results in the studies.

### Presentation of evidence mapping

We provided a scoping review and mapping evidence through a descriptive table that consisted of the characteristics of selected studies. The narrative description was presented.

## Results

### Study selection

**Fig. 1:**
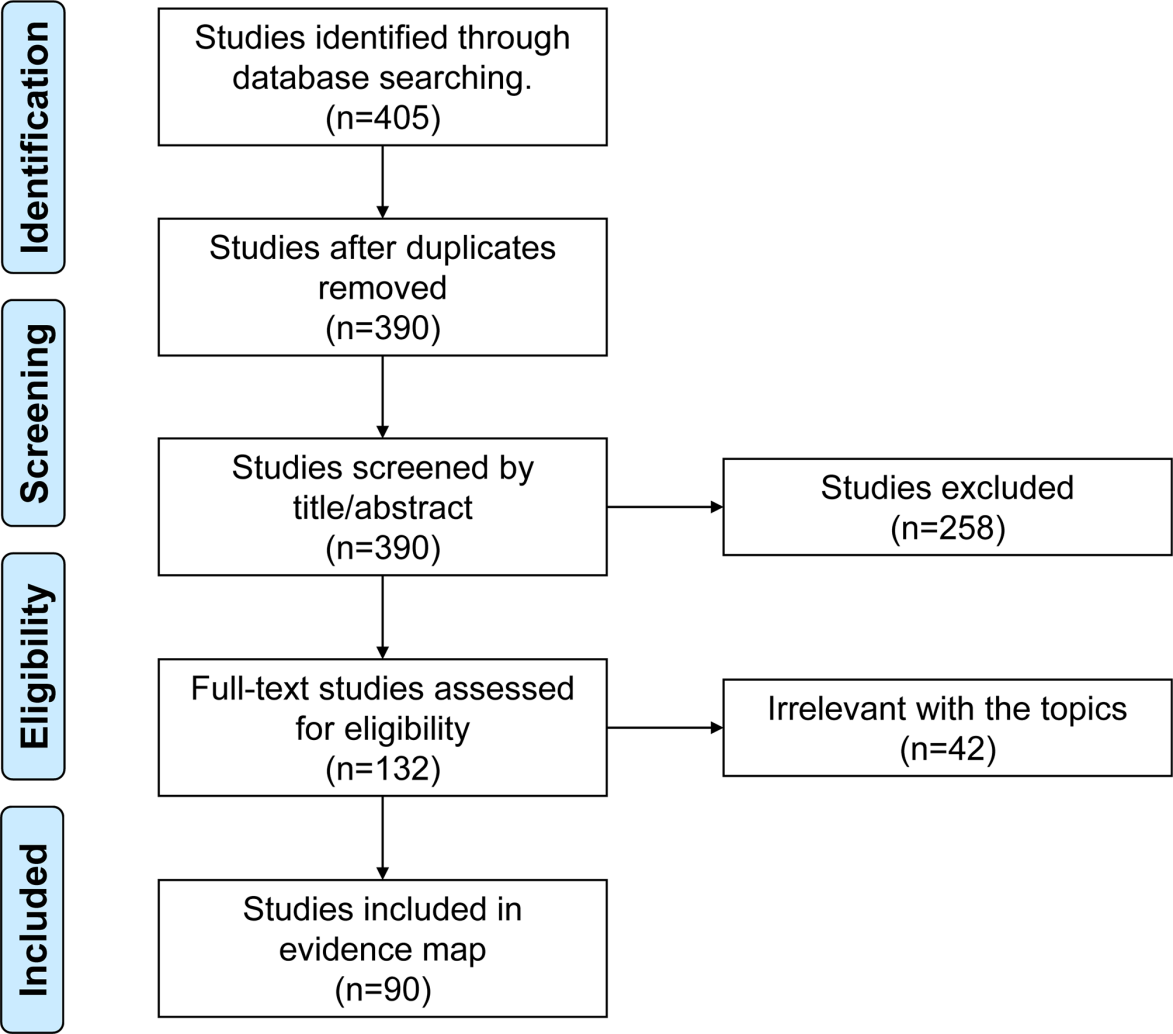
PRISMA flow diagram

A primary search yielded a total of 405 studies. After the removal of 15 duplicated publications, 390 studies were subsequently screened. Subsequently, full texts of 132 studies were scrutinized for eligibility. Ultimately, 90 studies met the eligibility criteria for inclusion in the scoping review and mapping evidence, comprising 2 phase I trials, 22 phase II trials, 2 phase III studies, 5 prospective studies, 36 retrospective studies, and 23 reviews (see Fig. 1).

### Could BEV-containing treatment regimens bring survival benefits to patients with rGBM, compared with non-BEV treatment regimens?

**Table 1:** The therapeutic efficacy of BEV-containing treatment regimens and non-BEV treatment regimens

Study	Study type	Diagnosis	Sample size (%)	Treatment arm	Primary endpoint and effect size	Key Findings	Reference
2010, Moen et al.	Review	rGBM	NA	NA	No pooled data	BEV-containing regimen improved ORR and PFS. Further study is needed to see the improvement of OS.	[57]
2010, Chamberlain et al.	Review	rGBM	NA	BEV-containing regimen	No pooled data	The improved ORR that was observed in the BRAIN and NCI 06-C-0064E studies resulted in the accelerated approval of single-agent BEV for patients with progressive GBM after previous TMZ-based therapy.	[58]
2012, Park et al.	Retrospective study	rGBM	11 (100%)	BEV + GKSR + Chemotherapy vs. GKSR + Chemotherapy	**Median OS**: BEV + GKSR + Chemotherapy vs. GKSR + Chemotherapy: 17.9 vs. 12.2 months (P = 0.005)	OS favored BEV plus GKSR plus chemotherapy group.	[59]
2014, Taal et al.	Phase II trial	rGBM	148 (100%)	Bevacizumab vs. Lomustine vs. BEV/LOM 110 vs. BEV/LOM 90	**9-month OS**: BEV vs. LOM vs. BEV/LOM 110 vs. BEV/LOM 90: 38% (95%CI 25–51) vs. 43% (95%CI 29–57) vs. 87% (95%CI 39–98) vs. 59% (95%CI 43–72) **12-month OS**: BEV vs. LOM vs. BEV/LOM 110 vs. BEV/LOM 90: 26% (95%CI 15–39) vs. 30% (95%CI 18–44) vs. 63% (95%CI 23–86) vs. 45% (95%CI 30–59) **Median OS**: BEV vs. LOM vs. BEV/LOM 110 vs. BEV/LOM 90: 8 months (95%CI 6–9) vs. 8 months (95%CI 6–11) vs. 16 months (95%CI 2–34) vs. 11 months (95%CI 8–12)	BEV plus LOM could prolong OS of rGBM compared with LOM single-agent.	[13]
2014, Khasraw et al.	Review	rGBM	NA	BEV-containing regimen vs. non-BEV regimen	No pooled data	Only one randomized study addressed the efficacy of bevacizumab in the recurrent setting.	[60]
2014, Larson et al.	Review	rGBM	55 (17.1%)	BEV + GKSR vs. GKSR	**Median OS (since diagnosis)**: BEV + GKSR vs. GKSR: 33.2 months (95%CI 23.7–42.7) vs. 26.7 months (95%CI 21.8–31.6)	BEV plus GKSR prolonged the OS in patients with rGBM compared with GKSR.	[35]
2014, Chauffer et al.	Phase II trial	rGBM	120 (100%)	TMZ + RT vs. BEV + IRI + RT + TMZ	**Median OS**: TMZ + RT vs. BEV + IRI + RT + TMZ: 11 months (95%CI 9–15) vs. 11 months (95% CI 9–15)	No significant difference	[61]
2016, Tosoni et al.	Review	rGBM	NA	NA	No pooled data	The efficacy of BEV raised controversy because of the lack of survival benefits.	[62]
2016, Balana et al.	Phase II trial	rGBM	55 (100%)	BEV + TMZ vs. TMZ	**Median OS**: BEV + TMZ vs. TMZ: 10.6 months (95% CI 6.9–14.3) vs. 7.7 months (95% CI 5.4–10.0) **HR for OS**: BEV + TMZ HR = 0.68 (95% CI 0.44–1.04, P = 0.07)	No significant difference	[63]
2016, Sánchez et al.	Retrospective study	rGBM	77 (100%)	BEV + Lomustine vs. non-BEV regimen	**Median OS (from diagnosis)**: BEV + Lomustine vs. non-BEV regimen: 17.63 (95% CI 15.38–19.89) vs. 13.23 months (95% CI 11.79–14.68, p = 0.049)	BEV-containing regimen prolonged the OS.	[64]
2017, Wick et al.	Phase III trial	rGBM	437 (100%)	BEV + LOM vs. LOM	**Median OS**: BEV + LOM vs. LOM: 9.1 months (95% CI 8.1–10.0) vs. 8.6 months (95% CI 7.6–10.4) **HR for OS**: BEV + LOM HR = 0.95 (95% CI 0.74–1.21; P = 0.65)	No significant difference	[9]
2017, Lombardi et al.	Review	pGBM and rGBM	4330 (100%)	BEV containing regimen vs. non-BEV regimen	**HR for OS**: BEV monotherapy HR = 1.09 (p = 0.7) BEV combined therapy HR = 0.96 (p = 0.3)	BEV treatment showed no benefits for OS but PFS.	[65]
2017, Hundsberger et al.	Review	rGBM	NA	NA	No pooled data	Treatment responses of rGBM with TMZ, LOM, and BEV and their combinations are short-lasting and did not show substantial survival advantages in randomized clinical trials.	[49]
2018, Wick et al.	Review	GBM and rGBM	NA	BEV + LOM vs. LOM	No pooled data	Many practicing clinicians described the positive effect of BEV plus LOM on PFS, other palliative effects, and neurological improvement in many patients as meaningful benefits, without OS gain in the entire patient population.	[33]
2018, Reardon et al.	Phase II trial	rGBM	48 (100%)	BEV + TBN vs. TBN	NA	No significant difference	[66]
2018, Carter et al.	Retrospective study	rGBM (first recurrence)	51 (16.6%)	BEV monotherapy vs. No treatment	**Median OS**: BEV monotherapy vs. no treatment: 15.4 months vs. 6.8 months (P = 0.00015)	Patients who received BEV treatment had a longer OS.	[67]
2018, Ameratunga et al.	Review	GBM and rGBM	3743 (100%)	Antiangiogenic therapy (one study didn’t use BEV) vs. non-antiangiogenic therapy	**HR for OS**: Antiangiogenic therapy vs. non-antiangiogenic therapy: HR = 0.99 (95% CI 0.85–1.16, P = 0.90)	Antiangiogenic therapy could not improve OS for rGBM significantly.	[68]
2019, Nguyen et al.	Retrospective study	First recurrent glioblastoma (GBM)	168 (100%)	BEV vs. LOM (2001–2004; 2009–2015) vs. BEV + LOM	**Median OS**: BEV vs. BEV + LOM vs. LOM 01–04 vs. LOM 09–15: 6.94 months vs. 7.13 months vs. 5.65 months vs. 14.1 months	No significant difference was observed between the BEV-containing regimen and the non-BEV groups. But subgroup analysis showed that BEV might be beneficial for rGBM patients with large tumor burden.	[15]
2019, Kim et al.	Review	rGBM	NA	NA	No pooled data	The concurrent approach with TMZ or BEV did not improve the OS of re-RT.	[69]
2019, Brandes et al.	Phase II trial	rGBM	123 (100%)	BEV + LOM vs. LOM	**Median OS**: BEV + LMS vs. LMS: 6.4 months vs. 5.5 months **HR for OS**: BEV + LOM HR = 1.04 (95% CI 0.69–1.59)	No significant difference	[70]
2020, Huang et al.	Prospective study	rGBM	22 (68.2%)	Surgery + BEV + Vincristine + Carboplatin vs. Surgery	**Median OS**: Surgery + BEV + Vincristine + Carboplatin vs. Surgery 13.5months (95% CI 6.5–89.3) vs. 3.2 months (95% CI 0.7–14.8; P = 0.006)	BEV-containing regimen prolonged OS of rGBM after surgery.	[71]
2020. Patel et al.	Prospective study	rGBM (large tumor burden)	67 (79.1%)	BEV containing regimen vs. surgery	**Median OS**: surgery vs. BEV-containing regimen 7.6 months vs. 4.3 months (P = 0.0376) **HR for OS**: BEV-containing regimen HR = 1.02 (95% CI 1.01–1.04, P = 0.009)	No significant difference	[44]
2020, Reardon et al.	Phase III trial	rGBM (first recurrence)	347 (47.6%)	Nivolumab vs. BEV	**Median OS**: Nivolumab vs. BEV: 9.8 months (95% CI, 8.2–11.8) vs. 10.0 months (95% CI, 9.0-11.8) **HR for OS**: BEV HR = 1.04 (95% CI, 0.83–1.30; P = 0.76)	There was no OS difference between nivolumab and BEV treated groups.	[72]
2020, Roth et al.	Review	GBM and rGBM	NA	NA	No pooled data	1. The addition of BEV to lomustine in patients with rGBM prolonged PFS but not OS. 2. BEV remains a useful option in patients with symptomatic tumors who experience a clinical benefit due to relief of the mass effect.	[73]
2020, Seystahl et al.	Retrospective study	rGBM (first recurrence)	344 (100%)	Alkylating agents + BEV vs. Alkylating agents	**Median OS (since the first recurrence)**: 1. Model 1 Alkylating agents vs. Alkylating agents + BEV 6.9 months (95% CI 5.3–8.5) vs. 7.1 months (95% CI 5.2–9.1) 2. Model 2 Alkylating agents vs. Alkylating agents + BEV 11.1 months (95% CI 10.2–12.1) vs. 7.4 (95% CI 5.7-9.0)	No benefits were observed from adding BEV to alkylating agents.	[41]
2020, Tan et al.	Review	GBM and rGBM	NA	NA	No pooled data	BEV could not improve OS but QoL with decreased corticosteroid use and thus sometimes is reserved for symptomatic patients at later recurrences.	[74]
2020, Hofmann et al.	Retrospective study	rHGG	61 (100%)	BEV-containing regimen vs. non-BEV regimen	**Median OS**: BEV vs. non-BEV: 10.3 months vs. 4.2 months (P = 0.023)	BEV prolonged OS of rGBM, especially in case of a second or later recurrence.	[75]
2021, Yamaguchi et al.	Retrospective study	rGBM	124 (100%)	Cytoreductive surgery + BEV vs. cytoreductive surgery	**Median OS (since the first recurrence)**: cytoreductive surgery + BEV vs. cytoreductive surgery: 16.3 months vs. 8.1 months (P = 0.007)	The addition of BEV to cytoreductive surgery prolonged OS since the first recurrence.	[27]
2021, McBain et al.	Review	rGBM	1734 (100%)	BEV-containing regimen vs. non-BEV regimen	**HR for OS**: BEV + LOM vs. LOM: No difference (HR = 0.91, 95% CI 0.75–1.10, moderate-certainty evidence) BEV vs. LOM: No difference (HR = 1.22, 95% CI 0.84–1.76, low-certainty evidence) BEV + IRI vs. LOM (HR = 1.16, 95%CI 0.71–1.88, very low-certainty evidence)	No significant difference	[39]
2021, Lovo et al.	Retrospective study	rGBM	46 (26.1%)	SRS + Chemotherapy (12BEV + 3TMZ) vs. SRS	**Median OS (since SRS)**: SRS + chemotherapy vs. SRS: 12 months vs. 7 months (P = 0.04)	BEV-containing regimen prolonged OS of patients with rGBM after SRS.	[76]
2021, Guan et al.	Retrospective study	rHGG	70 (50%)	HSRS + TMZ vs. HSRS + BEV vs. HSRS + BEV + TMZ vs. HSRS + BSC	**1-year OS**: BVZ + HSRS vs. HSRS alone: 77.3% vs. 56.0% (P = 0.035)	BEV treatment might be beneficial to HSRS treated rHGG patients.	[77]

Abbreviations: BEV, bevacizumab; BSC, best supportive care; GBM, glioblastoma; GKSR, Gamma Knife stereotactic radiosurgery; HSRS, hypofractionated stereotactic radiosurgery; LMS, lomustine; LOM, lomustine; ORR, objective response rates; OS, overall survival; PFS, progression-free survival; rGBM, recurrent glioblastoma; rHGG, recurrent high-grade glioma; SRS, stereotactic radiosurgery; TMZ, temozolomide;

In total, 31 studies (2 phase III studies, 5 phase II studies, 2 prospective studies, 9 retrospective studies, and 13 reviews, see Table 1) compared the therapeutic efficacy of BEV-containing treatment regimens with non-BEV treatment regimens. Of these, 17 studies investigated the benefits of adding BEV to chemotherapy (1 phase III trial, 1 phase II trial, 4 retrospective studies, and 11 reviews), while 5 studies investigated the efficacy of BEV plus lomustine (1 phase III trial, 3 phase II trial, and 1 retrospective study). Although a phase II study by Taal et al. showed the OS benefits of BEV plus lomustine versus the lomustine monotherapy group (mOS: LOM vs. BEV/LOM 110 vs. BEV/LOM 90, 8 months vs. 16 months vs. 11 months) [13], the other four randomized studies (including a phase III trial by Wick et al.) didn’t support this finding. Other phase II/III trials did not identify the OS benefits of BEV with a range of other chemotherapy partners (temozolomide (TMZ), trebananib, irinotecan, and nivolumab) compared with the non-BEV regimen.

Four studies (3 retrospective studies and 1 review) reported that the combination of BEV and radiotherapy improved OS, compared with radiotherapy alone. Meanwhile, 1 retrospective study and 1 prospective study on BEV plus re-surgery regimen showed that rGBM patients benefitted from BEV after receiving re-surgery.

Although some randomized clinical trials showed positive effects of the BEV-containing regimen on PFS, other palliative effects, and neurological improvement as meaningful benefits, gain on OS was not observed among the entire patient population in the majority of the trials.

### Could BEV combined therapy prolong the OS of patients with rGBM compared with BEV monotherapy?

Because BEV treatment alone lacked evidence to prolong OS of patients with rGBM, 41 studies were further conducted to identify the optimal combination therapies. These studies included 14 phase II trials, 1 phase I trial, 15 retrospective studies, 1 prospective study, and 10 reviews (Table 2).

**Table 2:** The therapeutic efficacies of BEV monotherapy and combined therapy

Study	Study type	Diagnosis	Sample size (%)	Treatment arm	Primary endpoint and effect size	Key findings	Reference
2009, Welch et al.	Review	rGBM	NA	BEV + IRI vs. BEV monotherapy	**Median OS**: BEV + IRI vs. BEV: 8.9 months vs. 9.7 months	BEV plus IRI only showed a slight gain of survival (9.7 vs. 8.9 months), versus 30 weeks (7–8 months) for historical controls.	[19]
2009, Friedman et al.	Phase II trial	rGBM	167 (100%)	BEV vs. BEV plus IRI	**Median OS**: BEV vs. BEV plus IRI: 9.2 months (95% CI 8.2–10.7) vs. 8.7 months (95% CI 7.8–10.9)	BEV alone or in combination with IRI was well tolerated and active in rGBM but had no benefits on OS.	[78]
2012, Chinnaiyan et al.	Phase I trial	rGBM	19 (100%)	BEV + Vorinostat + IRI	**Median OS**: 7.3 months	PFS and OS were favored with a high dose of vorinostat combined with BEV plus IRI.	[79]
2012, Johansson et al.	Review	rGBM (first recurrence)	NA	BEV + IRI vs. BEV monotherapy	**Median OS**: BEV + IRI vs. BEV: 8.7 months vs. 9.2 months	No OS benefits were observed in BEV plus IRI group.	[80]
2013, Weller et al.	Review	rGBM	NA	BEV combined therapy vs. BEV monotherapy	No pooled data	Combination regimens did not produce evidence of superior activity but commonly produced more toxicity.	[81]
2014, Clark et al.	Retrospective study	rGBM	18 (85.7%)	BEV + HSRS + plus chemotherapy	**Median OS**: 12.5 months	BEV plus SRS might improve the prognosis of rGBM.	[26]
2014, Taal et al.	Phase II trial	rGBM	148 (100%)	BEV vs. LOM vs. BEV/LOM 110 vs. BEV/LOM 90	**9-month OS**: BEV vs. LOM vs. BEV/LOM 110 vs. BEV/LOM 90: 38% (95% CI 25–51) vs. 43% (95% CI 29–57) vs. 87% (95% CI 39–98) vs. 59% (95% CI 43–72) **12-month OS**: BEV vs. LOM vs. BEV/LOM 110 vs. BEV/LOM 90: 26% (95%CI 15–39) vs. 30% (95% CI 18–44) vs. 63% (95% CI 23–86) vs. 45% (95% CI 30–59) **Median OS**: BEV vs. LOM vs. BEV/LOM 110 vs. BEV/LOM 90: 8 months (95% CI 6–9) vs. 8 months (95% CI:6–11) vs. 16 months (95% CI 2–34) vs. 11 months (95% CI 8–12)	BEV plus LOM prolonged OS of patients with rGBM compared with BEV monotherapy.	[13]
2014, Soffietti et al.	Phase II trial	rGBM	54 (100%)	BEV + Fotemustine	**Median OS**: BEV + Fotemustine: 9.1 months (95% CI 7.3–10.3)	BEV plus fotemustine combined therapy was not superior to either BEV or fotemustine monotherapy.	[82]
2015, Wong et al.	Retrospective study	rGBM (first recurrence)	37 (100%)	Novo TTF-100 A + BEV + TCCC vs. Novo TTF-100 A + BEV	**Median OS**: Novo TTF-100 A + BEV + TCCC vs. Novo TTF-100 A + BEV: 10.3 months (95% CI 7.7–13.6) vs. 4.1 months (95% CI 0.3–22.7; P = 0.0951)	no significant difference	[83]
2015, Wu et al.	Phase II trial	rGBM	73 (100%)	BEV monotherapy vs. BEV + vorinostat	**Median OS**: BEV + vorinostat vs. BEV: 9.2 months vs. 7.9 months, P = 0.75)	no significance difference	[84]
2015, Puduvalli et al.	Phase II study	rGBM	83 (100%)	BEV + vorinostat vs. BEV monotherapy	**Median OS**: BEV + vorinostat vs. BEV (8.3 vs. 7.0 months; P = 0.93)	no significant difference	[85]
2015, Matsuoka et al.,	Review	rGBM	NA	BEV monotherapy vs. BEV combined therapy	No pooled data	Neither BEV monotherapy nor BEV combined therapy showed to prolong OS.	[34]
2015, Galanis et al.	Phase II study	rGBM	121 (100%)	BEV + Dasatinib vs. BEV + PLA	**Median OS**: BEV + Dasatinib vs. BEV + PLA: 7.2 months vs. 7.9 months **HR for OS**: BEV + Dasatinib HR = 0.86 (95% CI 0.56–1.31, P = 0.48)	No significant difference	[86]
2015, Field et al.	Phase II trial	rGBM	120 (100%)	BEV + Carboplatin vs. BEV monotherapy	**Median OS**: BEV + Carboplatin vs. BEV: 6.9 months vs. 7.5 months **HR for OS**: BEV + carboplatin HR = 1.18 (95% CI 0.82–1.69, P = 0.38)	No significant difference	[87]
2016, Weathers et al.	Phase II trial	rGBM	49 (100%)	BEV + LOM vs. BEV monotherapy	**Median OS**: BEV + LOM vs. BEV: 13.05 months (95% CI 7.08–17.82) vs. 8.79 months (95% CI 6.42–20.22)	No significant difference	[14]
2016, Peng et al.	Retrospective study	rGBM	63 (100%)	BEV vs. BEV + valganciclovir	**Median OS**: BEV vs. BEV + valganciclovir: 8.7 months (95% CI 6.8–10.8) vs. 13.1 months (95% CI 9.13-NA) **HR for OS**: HR = NA (log-rank P = 0.005)	Valganciclovir in combination with BEV prolonged OS, compared with BEV monotherapy.	[21]
2016, Heiland et al.	Retrospective study	rGBM	35 (100%)	BEV monotherapy vs. BEV + LOM	**Median OS**: BEV alone vs. BEV + LOM: 4.07 months (95% CI 3.02–12.98) vs. 6.59 months (95% CI 5.51–16.3; P = 0.0238) **HR for OS**: BEV + LOM HR = 0.43 (95% CI 0.2–0.95).	BEV plus LOM prolonged the OS of patients with rGBM.	[16]
2017, Gilbert et al.	Phase II trial	rGBM	123 (100%)	BEV + TMZ vs. BEV + IRI	**Median OS**: BEV + TMZ vs. BEV + IRI: 9.4 months (95% CI 6.7–10.7) vs. 7.7 months (95% CI 6.7–9.1)	No significant difference	[88]
2017, Cloughesy et al.	Phase II trial	rGBM (first recurrence; BEV naive)	129 (100%)	BEV + ONA vs. BEV + PLA	**Median OS**: BEV + ONA vs. BEV + PLA: 8.8 months vs. 12.6 months **HR for OS**: BEV + ONA HR = 1.45 (95% CI 0.88–1.37; P = 0.1389) **9-months OS**: BEV + ONA vs. BEV + PLA: 49.7% vs. 57.2% (P = 0.4115)	No significant difference	[40]
2017, Birk et al.	Review	rHGG	NA	NA	No pooled data	BEV resulted in improvements in PFS in patients with rGBM secondary to microvascular regression, but improvements in OS were limited	[47]
2017, Azoulay et al.	Retrospective study	rGBM	180 (100%)	repeated surgery + salvage chemo and/or RT (containing BEV) vs. No repeated surgery + salvage chemo and/or RT (containing BEV) vs. repeated surgery alone vs. BSC	**Median OS**: repeated surgery + salvage chemo and/or RT (include BEV) vs. repeated surgery alone: 10 months vs. 6.8 months (P = 0.4727)	No significant difference	[28]
2017, Kesari et al.	Retrospective study	First recurrent glioblastoma	109 (52.9%)	TTF + BEV vs. BEV monotherapy	**Median OS**: TTF + BEV vs. BEV: 11.8 months vs. 9.0 months **HR for OS**: TTF + BEV HR = 0.61 (95% CI 0.37–0.99; P = 0.043)	TTF plus BEV prolonged OS, compared with BEV monotherapy.	[29]
2017, Hundsberger et al.	Review	rGBM	NA	NA	No pooled data	Treatment responses with TMZ, LOM, and BEV and their combinations were short-lasting and did not show substantial survival advantages in randomized clinical trials of rGBM.	[49]
2017, Diaz et al.	Review	GBM and rGBM	1249 (100%)	BEV monotherapy vs. BEV combined therapy	**Pooled median OS**: BEV monotherapy vs. BEV combined therapy: 31 to 40 weeks (weighted median OS: 36.2 ± 3.8 weeks, 95% CI 32.5–41.5) vs. 15 to 44.6 weeks (weighted median OS: 39.5 ± 6.2 weeks, 95% CI 39.5–44.8)	There was an observed increased OS when patients with recurrent GBM were treated with BEV alone or in combination with cytotoxic chemotherapy, compared with historical cytotoxic chemotherapy control.	[31]
2018, Song et al.	Review	rGBM	574 (100%)	combination group (BEV + LOM) vs. monotherapies group (BEV alone or LOM alone)	**OR for OS**: combination group vs. monotherapies group: OR = 0.84 (95% CI 0.68–1.03, P = 0.09)	LOM plus BEV was beneficial on PFS. But there was no advantage on OS	[18]
2018, Schernberg et al.	Retrospective study	rHGG	35 (100%)	BEV + reirradiation	**Median OS (since diagnosis)**: 44.6 months **Median OS (since reirradiation)**: 10.5 months (95% CI: 7.6–13.4)	1. Concomitant reirradiation with BEV was beneficial for rHGG patients. 2. BEV-naïve status was the only factor that was independently associated with improved OS (P = 0.002)	[25]
2018, Palmer et al.	Retrospective study	rHGG	118 (100%)	BEV + FSRS	**Median OS (since diagnosis)**: 26.7 months (95% CI 24.7–33.3, range 9.7-175.2) **Median OS (since recurrence)**: 13.8 months (95% CI 12.3–16.1, range 1.8–53.0).	The combination of FSRS and BEV for recurrent/progressive HGG provided promising results in terms of OS.	[24]
2018, Fat et al.	Retrospective study	rHGG	92 (100%)	BEV monotherapy vs. BEV + other chemotherapy	**12-months OS**: BEV + other chemotherapy vs. BEV monotherapy: 32% vs. 14% (P = 0.07)	No significant difference was observed between the BEV monotherapy and combined therapy groups.	[89]
2018, Bota et al.	Phase II trial	rGBM	8 (100%)	BEV + ERC1671 vs. BEV	**Median OS**: BEV + ERC1671 vs. BEV: 12 months vs. 7.5 months	No significant difference	[90]
2019, Morris et al.	Retrospective study	rGBM	45 (100%)	GKSR + BEV + chemotherapy	**Median OS (since diagnosis)**: 31.0 months (95% CI 18.6–39.4) **Median OS (since GKSR)**: 13.3 months (95% CI 7.4–24.9) after SRS	GKSR plus BEV was beneficial and safe.	[23]
2019, Nguyen et al.	Retrospective study	rGBM (first recurrence)	168 (100%)	Bev vs. LOM (2001–2004; 2009–2015) vs. BEV + LOM	**Median OS**: BEV vs. BEV + LOM vs. LOM 01–04 vs. LOM 09–15: 6.94 months vs. 7.13 months vs. 5.65 months vs. 14.1 months	1. No significant difference 2. Subgroup analysis showed that BEV might be beneficial for rGBM patients with a large tumor burden.	[15]
2019, Galanis et al.	Phase II study	rGBM	121 (100%)	BEV + DST vs. BEV	**Median OS**: BEV + DST vs. BEV: 7.3 months vs. 7.7 months	No significant difference	[91]
2020, Bergman et al.	Prospective study	rHGG (BEV resistant)	35 (100%)	BEV containing chemotherapy + FSRS vs. BEV containing chemotherapy	**Median OS**: BEV containing chemotherapy + FSRS vs. BEV containing chemotherapy: 7.2 months (95% CI 6.1–8.1) vs. 4.8 months (95% CI 1.7–7.6, P = 0.11)	FSRS plus BEV containing chemotherapy improved tumor local control and PFS but not OS.	[22]
2020, Lee et al.	Phase II trial	rGBM (first recurrence)	115 (100%)	BEV + trebananib vs. BEV monotherapy	**Median OS**: BEV + Trebananib vs. BEV monotherapy: 7.5 months (95% CI 6.8–10.1) vs. 11.5 months (95% CI 8.4–14.2) **HR for OS**: HR = 1.46 (95% CI 0.95–2.27; P = 0.09)	No significant difference	[92]
2020, Puduvalli et al.	Phase II trial	rGBM	74 (100%)	BEV + vorinostat vs. BEV monotherapy	**Median OS**: BEV vs. Bevacizumab + vorinostat: 9.26 (95% CI 5.88–11.37) vs. 7.79 (95% CI 5.06–9.63, P = 0.6398)	No significant difference	[93]
2020, Seystahl et al.	Retrospective study	rGBM (first recurrence)	51 (14.8%)	BEV + alkylating agents vs. BEV monotherapy	**Median OS (since recurrence)**: BEV + Alkylating agents vs. BEV: 9.4 months (95%CI 7.7–11.2) vs. 5.1 months (3.5–6.7, P < 0.001)	Alkylating agents have activity in recurrent glioblastoma, especially in the context of MGMT promoter methylation.	[41]
2021, Cardona et al.	Retrospective study	rGBM	15 (100%)	BEV + osimertinib	**Median OS**: BEV + osimertinib: 9.0 months (95% CI 3.9–14.0)	BEV plus Osimertinib had a long-lasting meaningful benefit to some rGBM subgroups.	[20]
2021, Chen et al.	Review	rGBM	NA	NA	No pooled data	Studies showed that BEV was effective in prolonging PFS and alleviating edema but had no effect on prolonging OS.	[94]
2021, Detti et al.	Retrospective study	rHGG	92 (100%)	BEV + chemotherapy vs. BEV monotherapy	**Median OS**: BEV vs. BEV + other chemotherapy: 9.4 months (7.7–13.4) vs. 8.9 months (95% CI 7.2–11.7)	No significant difference	[95]
2021, Zheng et al.	Review	rGBM	NA	NA	No pooled data	LOM was the only chemotherapy drug that improved the efficacy of BEV in rGBM.	[17]
2021, Yamaguchi et al.	Retrospective study	rGBM	73 (58.9%)	Cytoreductive surgery + BEV vs. BEV monotherapy vs. BSC	**Median OS (since the first recurrence)**: Cytoreductive surgery + BEV vs. BEV vs. BSC: 16.3 months; 7.4 months; 4.6 months (p = 0.0008)	BEV plus cytoreductive surgery improved OS compared with BEV monotherapy.	[27]

Abbreviations: BEV, bevacizumab; DST, dasatinib; FSRS, Fractionated Stereotactic Radiosurgery; GBM, glioblastoma; GKSR, Gamma Knife stereotactic radiosurgery; HSRS, hypofractionated stereotactic radiosurgery; IRI, Irinotecan; LOM, lomustine; ONA, Onartuzumab; OS, overall survival; PFS, progression-free survival; PLA, placebo; RT, radiotherapy; rGBM, recurrent glioblastoma; rHGG, recurrent high-grade glioma; SRS, stereotactic radiosurgery; TMZ, temozolomide; TTF, tumor treating field

A range of chemotherapy candidates was studied, including lomustine, ONA, celecoxib, vorinostat, dasatinib, valganciclovir, and trebananib. The phase II trials by Taal et al. and Weathers et al. did not find the OS benefits of the addition of lomustine to BEV [13, 14], while the results varied in the two retrospective studies [15, 16]. Although several studies found that BEV plus lomustine could prolong PFS, compared with BEV monotherapy, its benefits on OS warranted further validation [17, 18]. In addition to lomustine, the OS benefits of Irinotecan (IRI), osimertinib, and valganciclovir were reported in some retrospective studies [19–21]. But currently, no high-quality evidence from RCT was found to further verify their positive effect on OS.

Five studies investigated the efficacy of BEV plus radiotherapy versus BEV monotherapy (1 prospective study and 4 retrospective studies) on OS. Although the prospective study found no significant difference in OS between the combination group and monotherapy group [22], the other four retrospective studies stated that radiotherapy plus BEV improved the rGBM prognosis by enhancing OS [23–26].

A retrospective study by Yamaguchi et al. in 2021 showed that the BEV plus re-surgery improved OS (mOS, Cytoreductive surgery + BEV vs. BEV, 16.3 months vs. 7.4 months, p = 0.0008) [27] while another retrospective study in 2017 did not find any difference between BEV combination and single regimen groups [28].

As TTF has emerged as a promising technique for tumor therapy, the efficacy of TTF plus BEV was also elucidated. A post-analysis of the EF-14 trial demonstrated that the combination of BEV and TTF brought more OS benefits, compared with BEV alone (mOS, TTF + BEV vs. BEV: 11.8 months vs. 9.0 months, p = 0.043) [29].

The combinatory partners of BEV were widely studied, and some BEV combined therapies (especially with lomustine and radiotherapy) were proved to have superior efficacy to BEV monotherapy. But additional research is required to determine the optimal combination of treatment modalities.

**Fig. 2:**
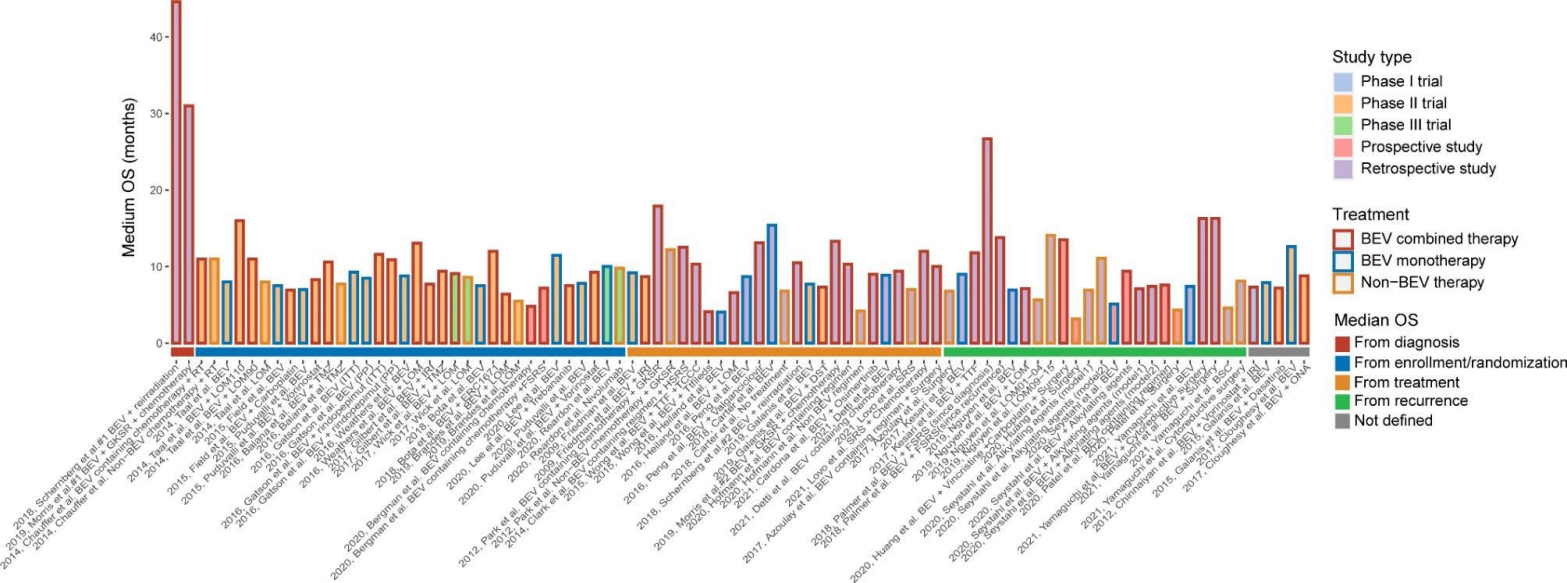
Median OS of patients with rGBM reported in studies

Median OS reported in the studies included in the analyses is summarized in Fig. 2. Although it was difficult to prove the OS benefits of BEV treatment through a single study, there was a trend to suggest that rGBM patients treated with BEV combined therapy may experience longer median OS.

### Could BEV treatment improve the quality of life and reduce the adverse events in rGBM?

In total, 19 studies (1 phase I trial, 4 phase II trials, 1 phase III trial, 4 retrospective studies, and 9 reviews) investigated the BEV effect on QoL and AEs (edema and cognitive dysfunction) (Table 3). While the effect of BEV monotherapy and combined therapy on OS prolongation remains unclear and controversial, three studies have verified BEV’s potential to reduce steroid use [30–32]. Additionally, three studies have reported that BEV could reduce the AEs induced by radiotherapy [33–35]. BEV also effectively controlled the tumor mass. However, only two retrospective studies found that the health-related QoL improved after receiving BEV containing therapy [30, 36], while other studies, including a phase II trial, did not find associations between BEV treatment and QoL [37, 38]. A review suggested that BEV combined therapy increased the incidence of side effects compared to BEV monotherapy [39]. Therefore, the potential for BEV to improve QoL remains uncertain and requires further validation.

**Table 3:** The effect of BEV on improving quality of life and reducing adverse events

Study	Study type	Diagnosis	Sample size	BEV treatment	Intervention	Key findings	Reference
2010, Vredenburgh et al.	Phase II trial	rGBM	167 (100%)	BEV combined therapy	BEV vs. BEV + IRI	A consistent reduction in median corticosteroid dose over time was found, relative to baseline.	[96]
2010, Keyrouz et al.	Phase II trial	rGBM	30 (100%)	BEV combined therapy	BEV + IRI	All patients had a clinical benefit and stopped taking steroids rapidly after starting BEV, regardless of radiological response.	[32]
2011, Nagpal et al.	Retrospective study	rGBM	20 (50%)	BEV combined therapy	BEV + chemotherapy vs. chemotherapy	BEV was beneficial for the independent living score, compared with the control group.	[30]
2014, Larson et al.	Review	rGBM	11 (3.4%)	BEV combined therapy	NA	BEV reduced detectable adverse radiation effects from 46–9% (P = 0.037)	[35]
2015, Matsuoka et al.	Review	rGBM	NA	BEV monotherapy and BEV combined therapy	NA	Discontinuation resulted in a rebound effect due to the loss of anti-edema properties.	[34]
2016, Mallick et al.	Review	rGBM	NA	BEV monotherapy and BEV combined therapy	NA	BEV alone or in combination also did not improve QoL.	[37]
2017, Wick et al.	Phase III trial	rGBM	437 (100%)	BEV combined therapy	BEV + LOM vs. LOM	The addition of bevacizumab to lomustine affected neither the health-related quality of life nor neurocognitive function.	[9]
2017, Diaz et al.	Review	GBM and rGBM	NA	BEV monotherapy and BEV combined therapy	NA	Despite the risks of therapy, the use of bevacizumab in the setting of rGBM clinically reduced the side effects of long-term steroid use in patients with rGBM.	[31]
2017, Badruddoja et al.	Phase II trial	rGBM	30 (100%)	BEV combined therapy	BEV + TMZ	No significant difference was observed in patients with rGBM treated with BEV of different cycles.	[38]
2018, Wick et al.	Review	GBM and rGBM	NA	BEV monotherapy and BEV combined therapy	NA	The beneficial effects on radionecrosis-related edema and neurological dysfunction were observed in many patients as meaningful benefits, in the absence of an overall survival gain in the entire patient population.	[33]
2018, Liu et al.	Retrospective study	Recurrent gliomas	20 (100%)	BEV combined therapy	BEV + TMZ	BEV treatment was beneficial to health-related quality of life compared with base level.	[36]
2018, Bent et al.	Phase II trial	Recurrent gliomas	155 (100%)	BEV combined therapy	BEV + TMZ vs. TMZ	No significant difference was observed between the two treatment groups.	[8]
2020, Tan et al.	Review	GBM and rGBM	NA	BEV monotherapy and BEV combined therapy	NA	BEV-containing regimen reduced the rates of radionecrosis.	[74]
2020, Matsuoka et al.	Retrospective study	rGBM	298 (100%)	BEV-containing regimens	BEV-containing regimens	The development of AEs to BEV-containing regimens was associated with unfavorable glioma-related survival outcomes in patients with rGBM.	[97]
2020, Roth et al.	Review	GBM and rGBM	NA	BEV monotherapy and BEV combined therapy	NA	BEV relieved the mass effect of GBM and rGBM.	[73]
2020, Korshoej et al.	Phase I trial	rGBM	15 (80%)	BEV monotherapy and BEV combined therapy	BEV vs. BEV + LOM	BEV administration reduced the steroid dose during the trial.	[98]
2021, McBain et al.	Review	rGBM	NA	BEV monotherapy and BEV combined therapy	NA	Receiving BEV containing regimen was associated with a higher frequency of SAEs compared with BEV monotherapy.	[39]
2021, Cardon et al.	Retrospective study	rGBM (EGFR amplification and EGFR vIII mutation)	14 (100%)	BEV combination	BEV + osimertinib	AEs with grade ≥ 2 were considered at least possibly related to osimertinib and BEV combination.	[20]
2021, Chen et al.	Review	rGBM	NA	BEV monotherapy and BEV combined therapy	NA	No significant difference was observed in the posttreatment quality of life or cognitive competence between the groups treated with or without BEV.	[94]

Abbreviations: AEs, adverse events; BEV, bevacizumab; FSRS, Fractionated Stereotactic Radiosurgery; GBM, glioblastoma; IRI, Irinotecan; LOM, lomustine; QoL, quality of life; rGBM, recurrent glioblastoma; SAEs, severity adverse events; TMZ, temozolomide

### Could subpopulations harboring some clinical or molecular characteristics gain survival benefits from BEV treatment?

A total of 17 studies (6 phase II trials, 2 prospective studies, and 9 retrospective studies) analyzed the types of rGBM that may favorably benefit from BEV-containing therapies. These studies analyzed the association between different genetic alterations, such as MGMT methylation, IDH mutation, and EGFR alteration and clinical features such as age groups, laboratory examinations, and radiological characteristics (Table 4).

**Table 4:** Responses to BEV in different rGBM subpopulations

Study	Study type	Diagnosis	Sample size (%)	BEV treatment	Intervention	Key findings	Reference
MGMT methylation status							
2014, Soffietti et al.	Phase II trial	rGBM	54 (100%)	BEV combined therapy	BEV + Fotemustine	MGMT promoter methylation was significantly associated with the improved PFS via univariate analysis.	[82]
2014, Taal et al.	Phase II trial	rGBM	132 (86.3%)	BEV-containing regimens and non-BEV regimens	BEV vs. Lomustine vs. BEV + Lomustine	PFS and overall survival were longer in patients with MGMT promoter methylated tumors.	[13]
2017, Badruddoja et al.	Phase II trial	rGBM	30 (100%)	BEV combined therapy	BEV + Temozolomide	No difference in the quality of life was observed between the unmethylated MGMT and methylated MGMT groups.	[38]
2017 Cloughesy et al.	Phase II trial	rGBM (first recurrence; BEV naive)	129 (100%)	BEV monotherapy and combined therapy	Bev + ONA vs. Bev + PLA	BEV plus ONA was recommended for rGBM with unmethylated MGMT while BEV monotherapy was recommended for rGBM with methylated MGMT.	[40]
2020, Seystahl et al.	Retrospective study	rGBM (first recurrence)	564 (100%)	BEV monotherapy and BEV plus alkylating agents	BEV + Alkylating agents	The difference of post recurrence survival was not significant between rGBM conditions with different MGMT statuses.	[41]
2021, Cardona et al.	Retrospective study	rGBM (EGFR amplification and EGFR vIII mutation)	14 (100%)	BEV combination	BEV + Osimertinib	PFS benefits from BEV combined therapy were observed in MGMT methylated rGBM.	[20]
IDH mutation status							
2011, Lv et al.	Retrospective study	rGBM	11 (17.5%)	BEV monotherapy and BEV combined therapy	BEV-containing regimen vs. non-BEV regimen	BEV-containing regimen improved OS and PFS of IDH mutated rGBM.	[43]
2014, Taal et al.	Phase II trial	rGBM	127 (83.0%)	BEV-containing regimens and non-BEV regimens	BEV vs. Lomustine vs. BEV + Lomustine	PFS and overall survival were both higher in IDH mutant tumors.	[13]
2021, Dono et al.	Retrospective study	rGBM (first recurrence)	43 (100%)	BEV combined therapy	BEV (administered in 81.4% patients) + SRS	IDH-WT rGBMs harboring PTEN mutation had a prolonged PFS and OS with BEV combined therapy.	[42]
EGFR alteration status							
2020, Reardon et al.	Phase II trial	rGBM (expressing EGFR vIII)	73 (100%)	BEV monotherapy and combined therapy	BEV + Rindopepimut vs. BEV	EGFR vIII-positive rGBM had a longer 6-month PFS, mOS, and 24-month OS after rindopepimut plus BEV treatment.	[72]
Age groups							
2009, Nghiemphu et al.	Retrospective study	rGBM	123 (100%)	BEV combined therapy	BEV containing regimen vs. non-BEV regimen	BEV treatment reflected a significant increase in PFS and OS, compared with the control group	[99]
2021, Barrascout et al.	Retrospective study	rGBM	47 (100%)	BEV monotherapy	BEV	Significant improvement based on the KPS scale was observed in non-elderly patients.	[48]
Laboratory examinations							
2016, Bertaut et al.	Retrospective study	rGBM	265 (100%)	BEV-containing regimen	BEV-containing regimen	Only patients with a high neutrophil count (> 6 G/L) benefited from the BEV-containing regimens.	[100]
2019 Quillien et al.	Prospective study	rGBM	29 (100%)	BEV monotherapy	BEV	Low neutrophil counts (< 3.9 G/L) and high Treg counts (above 0.011 G/L) had prolonged OS.	[46]
Radiological characteristics							
2014, Bahr et al.	Prospective study	rGBM	74 (100%)	BEV monotherapy	BEV	Double-positive (hyperintense lesions in T1 and diffusion-weighted restriction) rGBM patients had longer OS.	[45]
2017, Burger et al.	Retrospective study	rGBM	32 (100%)	BEV monotherapy and combined therapy	BEV vs. BEV + IRI vs. BEV + Lomustine	No survival benefits were observed between multifocal and resemble solitary GBMs.	[101]
2019, Nguyen et al.	Retrospective study	rGBM (first recurrence)	168 (100%)	BEV monotherapy and combined therapy	BEV vs. Lomustine vs. BEV + Lomustine	OS benefits from BEV were only observed in rGBM patients with a large tumor burden.	[15]
2020, Puduvalli et al.	Phase II trial	rGBM (large tumor burden)	67 (79.1%)	BEV monotherapy	BEV vs. Surgery	1. Pretreatment tumor volume was an independent risk factor for BEV treatment. 2. Large tumors with a low ADCL (lower apparent diffusion coefficient) benefitted from surgery, compared with BEV treatment.	[93]

Abbreviations: ADCL, apparent diffusion coefficient; BEV, bevacizumab; KPS, Karnofsky; ONA, Onartuzumab; OS; overall survival; PFS, progression-free survival; PLA, placebo; rGBM, recurrent glioblastoma;

### MGMT methylation status

MGMT methylation status was assessed in six studies (4 phase II trials and 2 retrospective studies) to determine its association with responses to BEV [38, 40, 41]. A phase II trial found that BEV plus ONA improved OS in patients with rGBM having unmethylated MGMT (mOS, ONA + BEV vs. PLA + BEV, 10.9 vs. 7.5 months, p = 0.0836), compared with BEV plus placebo while BEV monotherapy favored outcome in patients with rGBM harboring methylated MGMT (mOS, ONA + BEV vs. PLA + BEV, 7.7 months vs. NR, p = 0.0150) [40]. A retrospective study on BEV plus osimertinib treatment was marginally effective in most GB patients with simultaneous EGFR amplification plus EGFRvIII mutation [20]. Another retrospective study compared the post-recurrence survival between patients with MGMT methylation and unmethylation, treated with BEV plus alkylating agents and found no difference between the two groups [41]. Nevertheless, another phase II trial did not find differences in QoL between the groups with GBM having MGMT methylation and unmethylation to BEV plus TMZ [38].

### IDH mutation status

The association between IDH mutation status and response to BEV has been investigated in one phase II trial and two retrospective studies. Subgroup analysis of the BELOB trial revealed that patients with IDH mutation had higher OS and PFS compared to the control (mOS, IDH mutant vs. IDH wildtype: 20 vs. 9 months, p = 0.021) [13]. Dono et al. revealed an association between the genetic alterations and response to stereotactic radiosurgery (SRS) and BEV-containing chemotherapy in patients with rGBM carrying IDH-wildtype. Moreover, PTEN mutant subgroup in IDH WT group was found to have longer PFS and OS after combination therapy (mOS, PTEN mutant vs. PTEN wildtype: 22.5 vs. 13.6 months, p = 0.07; mPFS, PTEN mutant vs. PTEN wildtype: 17.5 vs. 8.1 months, p = 0.04) [42]. A retrospective study conducted by Lv et al. revealed that rGBM carrying IDH mutation had a better prognosis (OS and PFS) after receiving a BEV-containing regimen, compared with rGBM without IDH mutation (BEV monotherapy, mOS, IDH mutant vs. IDH wildtype: 10.16 vs. 4.9 months; mPFS, IDH mutant vs. IDH wildtype: 3.23 vs. 1.37 months, p = 0.04; BEV plus sunitinib, mOS, IDH mutant vs. IDH wildtype: 7.53 vs. 4.83 months; mPFS, IDH mutant vs. IDH wildtype: 2.07 vs. 1.10 months, p = 0.06), while no difference was found between IDH wildtype and mutated rGBM receiving non-BEV regimens (cetuximab and sunitinib) [43].

### EGFR alteration status

A phase II trial found that EGFR vIII positive rGBM had PFS and OS benefits from BEV plus rindopepimut therapy (HR for BEV plus rindopepimut, 0.58, p = 0.01).

### Radiological characteristics

Apart from genetic alterations, the association between radiological examination outcome and response to BEV was elucidated. Cox regression analysis in a phase II trial showed that BEV improved survival in patients with large enhancing tumors with low apparent diffusion coefficient (ADCL). It also revealed that the pretreatment tumor volume was an independent risk factor for the BEV-treated group [44]. A prospective study revealed that patients with hyperintense lesions in T1 and diffusion-weighted restriction (double-positive) benefited more than others from BEV treatment [34, 45]. A retrospective study demonstrated that rGBM with a large tumor burden might be benefitted most favorably from BEV-containing regimens [15].

### Laboratory examinations

A prospective trial in 2019 stated that low neutrophil counts (below 3.9 G/L) and high Treg counts (above 0.011 G/L) predicted prolonged OS [46].

### Age groups

No consensus was found regarding the association between BEV efficacy and age groups. Two retrospective studies found that there was a better improvement in non-elderly patients with rGBM/recurrent high-grade glioma (rHGG) patients compared with elderly patients treated with BEV-containing regimens [47, 48]. However, another retrospective study concluded controversially that elderly patients had more prognostic benefits compared with younger patients [49].

### What are the optimal dosages and indications for BEV administration?

The optimal dosages and indications for BEV administration are still under investigation. In the US, the recommended dosage of BEV in the US is a 10 mg/kg intravenous infusion administered every 2 weeks. However, different studies (2 retrospective studies and 2 reviews, Table 5) have adopted varying dosages, and recent research has elucidated the optimal dosage. Two retrospective studies stated that lower doses were at least equal or even superior to the recommended doses [50, 51]. Two reviews had similar conclusions ^[37, 49]^. Although BEV at the recommended dose and lower dose exhibits equal efficacy on survival, influence on other outcomes such as QoL and side effects reduction needs further investigation.

**Table 5:** The optimal dosage and indication for the BEV treatment

Study	Study type	Diagnosis	Sample size	BEV administration	Intervention	Key findings	Reference
The optimal dosage of BEV							
2011, Lorgis et al.	Retrospective Study	rHGG	219 (100%)	BEV combined therapy	5 mg/kg/week vs. less than 5 mg/kg/week	Low BEV dose intensity was the most significant independent prognostic factor of survival.	[50]
2015, Levin et al.	Retrospective Study	rGBM	181 (100%)	BEV combined therapy	BEV combined therapy	Dosing BEV at half the standard dose (standard dose: 10 mg/kg every 2 weeks) for progressive/rGBM was not inferior to standard dosing.	[51]
2016, Mallick et al.	Review	rGBM	NA	BEV monotherapy and BEV combined therapy	5 mg/kg BEV vs. 10 mg/kg BEV vs. 15 mg/kg BEV	The meta-analysis found no difference in dose-response of BEV between 5 mg/kg and 10–15 mg/kg.	[37]
2017, Hundsberger et al.	Review	rGBM (first recurrence)	NA	BEV monotherapy and BEV combined therapy	Lower doses BEV vs. Recommended doses BEV	The outcome of lower doses of BEV was equal to or superior to the recommended dose in retrospective studies of recurrent malignant gliomas including GBM.	[49]
The optimal opportunity for BEV treatment							
2013, Sahebjam et al.	Retrospective Study	rGBM and recurrent anaplastic gliomas	27 (100%)	BEV monotherapy and BEV combined therapy	BEV + TMZ vs. TMZ + Procarbazine vs. LMS vs. IRI + TMZ + Procarbazine	No significant difference in OS was found when comparing the subpopulation who were treated with BEV after the first relapse and those treated after the second or later relapse.	[53]
2014, Piccioni et al.	Retrospective Study	rGBM	468 (100%)	BEV combined therapy	BEV combined therapy	Deferred use of bevacizumab was not associated with diminished efficacy.	[102]
2015, Matsuoka et al.	Review	rGBM	NA	BEV monotherapy and BEV combined therapy	NA	1. The optimal duration of bevacizumab therapy was not established. 2. BEV continuation led to the development of a more aggressive phenotype while discontinuation resulted in a rebound effect due to loss of anti-edema properties. 3. Some data suggested that continuation beyond initial progression modestly improved survival in patients with recurrent glioblastoma. 4. For those patients who progressed despite a bevacizumab-containing regimen rarely responded to the second bevacizumab-containing chemotherapeutic regimen.	[34]
2016, Schaub et al.	Retrospective Study	rGBM (treated with BEV)	174 (100%)	BEV monotherapy and BEV combined therapy	BEV + IRI vs. BEV	Early use of BEV prolonged OS.	[103]
2016, Balana et al.	Retrospective Study	Newly diagnosed GBM and rGBM	28 (100%)	BEV-containing regimen	BEV-containing regimen	The rGBM patients who responded previously to BEV and stopped before progression, obtained benefit from a second and even a third re-introduction of the drug but did not respond as well to second or third-line treatments with other drugs.	[63]
2017, Blumenthal et al.	Retrospective Study	rHGG	59 (100%)	BEV combined therapy	Pre-surgery BEV administration vs. Post-surgery BEV administration	1. No significant difference in median OS from initial diagnosis was found between the pre-surgery and post-surgery groups. 2. Median OS from recurrent surgery of pre-surgery BEV treated groups was longer than that of the post-surgery group.	[52]
2019, Prelaj et al.	Retrospective study	rGBM (first recurrence)	26 (100%)	BEV combined therapy	concomitant FTM/BEV vs. sequential FTM/BEV	No significant difference.	[104]
2020, Seystahl et al.	Retrospective study	rGBM (first recurrence)	344 (100%)	BEV combined therapy	Alkylators first and BEV at any further recurrence vs. BEV first and alkylators at further recurrence	OS benefits were observed in alkylators first and BEV at any further recurrence.	[41]

Abbreviations: BEV, bevacizumab; FTM, fotemustine; GBM, glioblastoma; IRI, Irinotecan; LMS, lomustine; OS, overall survival; rGBM, recurrent glioblastoma; rHGG, recurrent high-grade glioma; TMZ, temozolomide;

The window of opportunity for BEV treatment is also still under debate. Matsuoka et al. argued that the initiation of a treatment regimen containing BEV at first recurrence may improve prognosis. However, they also noted that BEV administration could lead to chemotherapy resistance and rapid progression in some cases [34]. Similar conclusions were made in other studies. A retrospective study found that BEV treatment before surgery might be beneficial for young and high-performance patients [52]. No significant difference in OS was identified between patients receiving BEV-containing regimens after the first relapse and the second relapse [53]. However, some studies concluded contrastingly. Funakoshi et al. found that BEV administration after recurrence (post-BEV) improved PFS and deterioration-free survival (DFS) than pre-recurrence BEV administration (pre-BEV) (mPFS, post-BEV vs. pre-BEV: 9.9 vs. 7.5 months, p = 0.0153; mDFS, post-BEV vs. pre-BEV: 13.8 vs. 8.5 months, p = 0.0046) [54]. Therefore, the optimal opportunity window of BEV treatment warrants further validation through future large-scale clinical trials. Table 5 summarizes the different findings across studies.

## Discussion

BEV has shown improved PFS in clinical studies, but OS benefits have not been consistently observed. Despite this, BEV has been proposed as a promising drug in GBM due to its ability to reduce side effects from steroid use and radiotherapy. To further maximize benefits from BEV treatment, investigations could be summarized in two ways. One was to combine BEV with other treatment modalities to enhance synergistic anti-tumor effects. The other one was to identify the BEV-response groups which could gain more prognostic benefits from the treatment of BEV. Additionally, we investigated the optimal dosage and treatment opportunity window to maximize the BEV treatment benefits. To the best of our knowledge, BEV-containing multimodality treatment was associated with clinical benefit and is worthy of administration. The outcome depends on the unique clinical and molecular features linked to varied BEV responses.

Despite many efforts in the past, the efficacy of BEV remains to be optimized and needs further investigations focusing on the two mechanisms mentioned above. First, newly emerging therapies for rGBM bring further opportunities for BEV-containing multimodality treatment. TTF was the landmark therapy in the treatment of GBM [55]. Post-hoc analysis of EF-14 in a phase III trial on newly diagnosed GBM revealed that the addition of TTF to BEV could further prolong the median OS by 2 months beyond the period that patients with rGBM achieved with second-line treatment alone [56]. Studies of higher evidence are warranted to investigate the efficacy of BEV plus TTF combination therapies. Besides TTF, an increasing number of combination therapies are currently explored via several clinical trials (e.g., NCT02511405, VB-111 plus BEV; NCT01308684, RO5323441 plus BEV; NCT01349660, BKM120 plus BEV).

Second, biomarker-enrichment strategies are warranted to direct the clinical administration of BEV. While BEV administration has been shown to improve OS in the TCGA-proneural newly diagnosed GBM subtype, characterizing rGBM according to TCGA transcriptome classification in a realistic manner requires further exploration. Moreover, high-quality evidence is lacking regarding the associations between molecular and clinical features with BEV response. Therefore, RCTs focusing on specific subpopulations of rGBM are warranted.

In summary, current RCTs are not sufficient to make a definitive statement that BEV could improve OS and QoL in patients with rGBM although some clinical benefits (including PFS, decreased steroid use, and cognitive ability protection) are observed. Combing BEV with TTF and administration at first recurrence may improve prognosis. In the meantime, rGBM with low ADCL, large tumor burden, or IDH mutation is more likely to benefit from BEV treatment. Of note, observational studies have yielded conflicting results due to heterogeneity. High-quality clinical trials are needed to gain new insights into BEV treatment, and breakthroughs may emerge from the use of BEV-containing multimodality treatment on unique subpopulations of rGBM.

## Data Availability

All data generated or analyzed during this study are included in this published article.

## Abbreviations

ADCL: Apparent diffusion coefficient
AEs: Adverse events
BEV: Bevacizumab
BSC: Best supportive care
CCT: Case-control trial
DFS: Deterioration-free survival
EANO: European Association for Neuro-Oncology
FDA: U.S. Food and Drug Administration
FSRS: Fractionated stereotactic radiosurgery
GBM: Glioblastoma
GKSR: Gamma Knife stereotactic radiosurgery
HSRS: Hypofractionated stereotactic radiosurgery
IRI: Irinotecan
KPS: Karnofsky Performance Status
MGMT: O6-methylguanine-DNA methyltransferase
mOS: Median OS
ORR: Objective response rates
OS: Overall survival
PFS: Progression-free survival
QoL: Quality of life
RCT: Randomized controlled trial
RT: Radiotherapy
rGBM: Recurrent glioblastoma
rHGG: Recurrent high-grade glioma
SAEs: Severe adverse events
SRS: Stereotactic radiosurgery
